# The impact of positive microbiology results on adherence to antimicrobial stewardship post-prescription review and feedback (PAF) rounds in a quaternary referral center

**DOI:** 10.1017/ash.2023.120

**Published:** 2023-03-02

**Authors:** Matthew D.M. Rawlins, Peter A. Boan

**Affiliations:** 1 Department of Pharmacy, Fiona Stanley Hospital, Murdoch, Western Australia; 2 Department of Infectious Disease, Fiona Stanley Hospital, Murdoch, Western Australia, Australia; 3 Department of Microbiology, PathWest Laboratory Medicine WA, Fiona Stanley Hospital, Murdoch, Western Australia, Australia

Guidelines for antimicrobial stewardship (AMS) programs suggest that preauthorization and postprescription review and feedback (PAF), ideally in combination, are optimal.^1^ Another key element of an AMS program is the microbiology laboratory.^1^

Microbiology laboratories have recently focused on the utility of rapid diagnostic testing^2^ along with the optimal provision of results to appropriately influence patient management.^3^ One method of delivering microbiology results is through AMS PAF rounds. However, to our knowledge, there has been no evaluation of the impact on adherence to PAF round advice of positive microbiology laboratory results compared with advice based solely on clinical diagnosis and trajectory.

Our Australian quaternary hospital has had an established AMS program since opening in 2014, incorporating daily PAF rounds, by an infectious diseases (ID) physician and AMS pharmacist for patients outside hematology and the intensive care unit, which have different arrangements.^4^ We evaluated the impact of culture data on adherence to the PAF recommendations of a well-established hospital AMS team. This audit was approved as an Institutional Quality Improvement Activity (no. 47840) and was exempt from requiring Human Research and Ethics approval.

## Methods

We utilized data collected as part of an established AMS program at a 783-bed hospital in Australia. PAF rounds are of 2–3 hours duration on weekdays by regular, rostered ID physicians together with the permanent full-time AMS pharmacist. PAF round responses typically occur within 24–48 hours of submission.^4^ Rounds are usually conducted in person and patients are reviewed once, with repeated reviews only in the setting of new information or diagnoses. PAF rounds primarily focus on reviewing restricted antimicrobials, including glycopeptides, carbapenems, β-lactamase combinations, third- and fourth-generation cefalosporins, and fluoroquinolones.^5^

The 4 categories of PAF advice were (1) “add” when the addition of an antimicrobial was needed to provide expanded spectrum; (2) “confirm” when the current regimen was appropriate; (3) “stop” when ceasing 1 or more antibiotics for lack of appropriateness or adequate duration; and (4) “substitute” for changing to broader or narrower spectrum per diagnosis, switch to directed therapy, or switch from intravenous to oral therapy (ie, IV to PO).

The AMS pharmacist entered adults reviewed on PAF rounds into a Microsoft Access Database (Microsoft, Redmond, WA). Periodic assessment to PAF adherence occurs annually for quality assurance at our institution.

The institutional microbiology laboratory uses matrix-assisted laser desorption–ionization-time of flight mass spectrometry (MALDI-TOF; Bruker Biotyper, Bruker Daltonik GmbH, Breman, Germany) including direct MALDI-TOF from flagged blood cultures. Susceptibility testing predominantly uses Vitek2 (bioMerieux, St. Louis, MO), with Kirby-Bauer direct-disc susceptibility testing for urine and flagged blood cultures. Cascading antimicrobial reporting is used, and all susceptibility results are available to the AMS team.^6^

Between October 1, 2014, and September 30, 2022, 12,886 PAF reviews were performed. We retrospectively reviewed our database and compared patients with or without positive microbiology. For this study, only reviews that had complete data on adherence to antimicrobial choice recommendations were included (N = 3,562). Comparison between groups was made using the χ^2^ analysis at the 0.05 level of significance.

## Results

Data from 3,562 PAF reviews (27.6%) were included: 2,157 in the empirical group and 1,405 in the positive microbiology group. Advice to stop antimicrobials was more common in the empirical group. Overall, adherence to advice for antimicrobial choice was higher for the positive microbiology group compared with the empirical group. We detected higher rates of adherence in the positive microbiology group for the substitute and stop categories, which was statistically significant for substitution. Adherence rates to advice were similar for adding and confirming antimicrobial prescriptions (Table 1).

**Table 1.** Proportion categories of recommendations and comparison of adherence to prospective audit and feedback (PAF) round advice on antimicrobial choice according to the presence of positive microbiology

Advice on antimicrobial choice	Empirical Proportion of advice provided (%)	Empirical Followed/total (%)	Positive microbiology proportion of advice provided (%)	Positive microbiology Followed/total (%)	Difference of advice followed Empirical versus Positive Microbiology (P:value)
Add	42/2157 (1.9)	37/42 (88.1)	47/1405 (3.3)	41/47 (87.2)	.904
Confirm	610/2157 (28.2)	601/610 (98.5)	457/1405 (32.5)	450/457 (98.5)	.9442
Substitute	814/2157 (37.7)	666/814 (81.8)	770/1405 (54.8)	682/770 (88.6)	**.00016**
Stop	691/2157 (31.5)	501/691 (72.5)	131/1405 (9.3)	105/131 (80.1)	.06876
Total	2157	1805/2157 (83.7)	1405	1278/1405 (91.0)	**<.00001**

Bold values are statistically significant at P < .05.

## Discussion

AMS programs are associated with reduced use of restricted antimicrobials and lower antimicrobial resistance.^1^ PAF review is 1 of the 2 core elements of AMS programs, especially targeting restricted broad-spectrum antimicrobials.^1^ Risk factors for nonadherence to PAF advice include the following: type of infection, disease severity, prolonged hospital admission, prior hospitalisation, as well as the specialty and experience of the managing clinician.^7^ Conversely, PAF advice is more likely to be followed with face-to-face feedback, such as with “handshake stewardship,” which is resource intensive but fosters a more collaborative relationship between the AMS team and frontline clinicians.^8^ Direct feedback during PAF rounds has built relationships with clinical teams and has increased adherence to our PAF advice.^5^

Overall, frontline clinicians appeared more willing to follow advice to continue or add than to substitute or stop antimicrobials. The concept of clinical inertia, in which clinicians are unwilling to change decisions made by prior prescribers, has been explored in the literature.^9^ Advice to stop antimicrobials was 5 times more frequent in the empirical group, probably due to diagnosis uncertainty at the time of the early PAF review.

The microbiology laboratory contributes to AMS program decision-support tools and institutional antibiogram development.^2^ PAF rounds require that laboratory results are accurate, significant, and timely, primarily for appropriate de-escalation of antimicrobial therapy. Advice for substitution or stopping was followed more where positive culture results were available. Thus, culture results can provide additional support to the expert opinion of an ID physician or AMS pharmacist.

This study had several limitations. The culture site, specific pathogen, antimicrobial resistance patterns, infection source, and/or patient factors may have contributed to the higher adherence rates in patients with positive microbiology results. We did not control for these factors, and they may have been confounders to the findings. Other limitations include the single quaternary centre, retrospective, observational review, which may not be generalisable to other settings. A qualitative survey of clinicians would be informative.

In conclusion, positive culture results were associated with better adherence to PAF advice. Communicating microbiology results through PAF rounds is a valuable AMS strategy.
